# Processive ATP-driven Substrate Disassembly by the *N*-Ethylmaleimide-sensitive Factor (NSF) Molecular Machine*[Fn FN3]^♦^

**DOI:** 10.1074/jbc.M113.476705

**Published:** 2013-06-17

**Authors:** Daniel J. Cipriano, Jaemyeong Jung, Sandro Vivona, Timothy D. Fenn, Axel T. Brunger, Zev Bryant

**Affiliations:** From the ‡ Department of Molecular and Cellular Physiology,; ** Department of Neurology and Neurological Science,; ‖ Department of Photon Science,; § Howard Hughes Medical Institute,; ‡‡ Department of Structural Biology, and; ¶ Department of Bioengineering, Stanford University, Stanford, California 94305

**Keywords:** ATPases, Exocytosis, Fluorescence, Membrane Fusion, Molecular Motors, AAA+ ATPase, Mechanochemical Coupling, Remodeling, Translocase, Unfoldase

## Abstract

SNARE proteins promote membrane fusion by forming a four-stranded parallel helical bundle that brings the membranes into close proximity. Post-fusion, the complex is disassembled by an AAA+ ATPase called *N*-ethylmaleimide-sensitive factor (NSF). We present evidence that NSF uses a processive unwinding mechanism to disassemble SNARE proteins. Using a real-time disassembly assay based on fluorescence dequenching, we correlate NSF-driven disassembly rates with the SNARE-activated ATPase activity of NSF. Neuronal SNAREs activate the ATPase rate of NSF by ∼26-fold. One SNARE complex takes an average of ∼5 s to disassemble in a process that consumes ∼50 ATP. Investigations of substrate requirements show that NSF is capable of disassembling a truncated SNARE substrate consisting of only the core SNARE domain, but not an unrelated four-stranded coiled-coil. NSF can also disassemble an engineered double-length SNARE complex, suggesting a processive unwinding mechanism. We further investigated processivity using single-turnover experiments, which show that SNAREs can be unwound in a single encounter with NSF. We propose a processive helicase-like mechanism for NSF in which ∼1 residue is unwound for every hydrolyzed ATP molecule.

## Teaser

**Background:** NSF is an AAA+ protein that recycles the post-fusion SNARE complex during the membrane fusion cycle.

**Results:** NSF disassembles the SNARE complex processively *in vitro* and consumes dozens of ATP molecules per SNARE.

**Conclusion:** NSF is a processive motor that progressively unwinds the SNARE complex.

**Significance:** The physical mechanism of this poorly understood machine is illuminated using new assays and new measurements of ATP coupling ratios and processivity.

## Introduction

ATPases associated with various cellular activities (AAA+ ATPases)^3^ couple the energy from ATP hydrolysis to a wide range of mechanical functions, including the disassembly and remodeling of macromolecular complexes. The hallmark feature of this superfamily is a structurally conserved ATPase domain (AAA+ domain) consisting of 200–250 amino acids, arranged in an oligomeric ring.

*N*-Ethylmaleimide-sensitive factor (NSF), one of the founding members of the AAA+ superfamily, plays a central role in the membrane fusion cycle. Membrane fusion events are facilitated by SNARE proteins, which contain highly conserved segments of heptad repeats, collectively termed the “SNARE motif.” In a SNARE complex, three or four SNARE proteins zipper together to form a parallel four-stranded helical bundle, anchoring the membranes in close proximity and driving the fusion process (1). Post-fusion, NSF harnesses the energy of ATP hydrolysis to disassemble the SNAREs, aided by the α-SNAP adapter protein. The individual SNAREs are then recycled into the vesicle pool. For recent reviews, see Refs. 2–5. Here we investigate the NSF-driven disassembly of the neuronal SNAREs that are involved in neurotransmitter release: syntaxin, synaptobrevin (also called VAMP, vesicle-associated membrane protein), and SNAP-25.

NSF is a homohexamer, consisting of protomers with an N-terminal adapter domain (NSF-N) and two AAA+ domains (NSF-D1 and NSF-D2). NSF-N is responsible for interacting with the SNARE substrate and the adapter protein α-SNAP. Positively charged residues on NSF-N have been shown to be required for α-SNAP-SNARE binding (6). NSF-D1 is believed to be the catalytically active ATPase domain and is involved in SNARE recognition. NSF-D2 is believed to be a structural scaffold that is responsible for nucleotide-dependent hexamerization, although hydrolysis stimulated by the D1 domain cannot be ruled out (7).

NSF has been studied since its discovery in 1988 (8), but the detailed molecular mechanism by which NSF disassembles SNAREs is unknown. ATP hydrolysis in NSF-D1 causes large conformational changes in NSF (9–11). Models for the coupling between these conformational changes and SNARE disassembly remain speculative. Crystal structures are available for the D2 domain (12, 13) and for the N-terminal domain (14, 15), but crystal structures of the full-length protein and the so-called 20S complex (NSF-SNAREs-α-SNAP) have been elusive.

**FIGURE 1.**
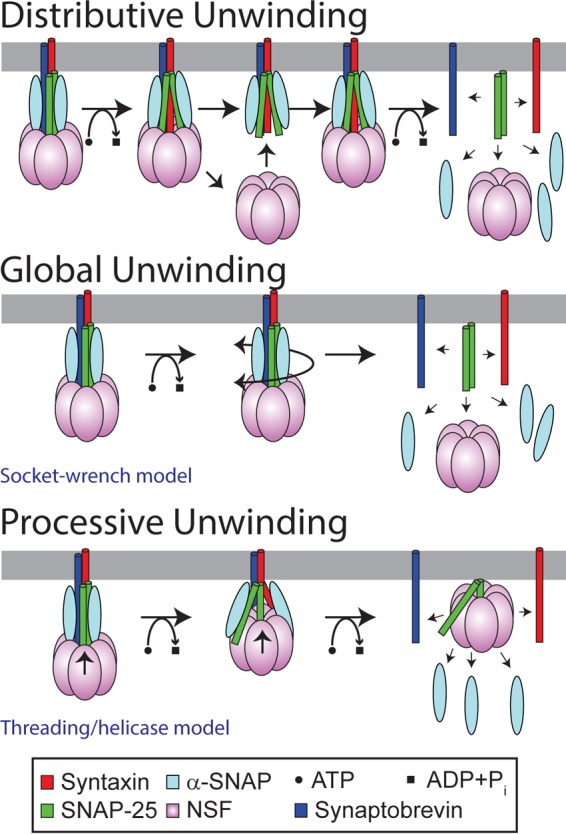
**Possible models for the action of NSF in SNARE disassembly.** For simplicity, the model only shows the core SNARE helices (and respective transmembrane domains), which are drawn as *cylinders*. Three classes of models are shown. Distributive unwinding involves multiple NSF binding and rebinding events, whereas processive and global unwinding involve the binding and action of a single NSF hexamer. Shown are specific examples of a global model (the socket-wrench model) and a processive model (the threading/helicase-like model) that have been proposed in the literature (16). In the socket-wrench model, conformational changes in NSF are transmitted through α-SNAP to apply a torque or force on the SNAREs that results in the global unwinding of the SNARE core. In the threading/helicase-like model, NSF unwinds the SNARES by the ATP-powered processive threading of one of the SNARE proteins through the pore of the hexamer. Note that the stoichiometry of the 20S complex is still uncertain; initial work suggested that three α-SNAP bound to one SNARE complex and one NSF (56). However, recent work (see Footnote 4) indicates that the initial α-SNAP-SNARE complex has a stoichiometry of 1:1, although this finding does not rule out the involvement of additional α-SNAPs in the 20S complex and during the disassembly reaction. For simplicity, the graphics in Fig. 1 are shown with three α-SNAPs.

In this work, we endeavor to distinguish between three broad classes of models for the action of NSF on the SNARE complex (Fig. 1). In a *distributive* model, complete unwinding of the SNARE complex requires many NSF binding and release events; a single binding event leads to only partial unwinding of the SNARE core, and the partially unwound complex then requires additional NSF binding events to complete the disassembly process. In a *global unwinding* model, NSF acts to destabilize the entire SNARE complex, whereas in a *processive unwinding* model, NSF advances stepwise from one end of the complex, progressively increasing the number of unwound residues until the entire complex is disassembled. The previously proposed “socket-wrench model” (10, 16, 17) is an example of global unwinding, and the “threading/helicase-like model” (16, 17) is an example of processive unwinding.

In the socket-wrench model, NSF and α-SNAP bind to the SNARE complex and apply a torque (or force) to the complex that results in global unwinding of the helical bundle (10, 16, 17). ATP-driven conformational changes in NSF-D1 are transmitted through NSF-N and α-SNAP, untwisting (or pulling on) the SNARE bundle and leading to disassembly.

In the threading/helicase-like model, NSF progressively pulls one of the SNARE protein components of the complex into the central cavity of the hexamer and thus destabilizes the SNARE core (16, 17). The model is analogous to the hexameric AAA+ helicases, which harness the energy of ATP hydrolysis to pull one DNA strand through the hexameric pore, thus separating double-stranded DNA (18–20). A number of homologous AAA+ proteins (ClpX, ClpA, FtsH, HslUV, and Lon) are peptide-translocating AAA+ proteins (17, 21). The best studied of these, ClpX, pulls its protein substrate through the central pore, unfolding the protein and feeding it into the ClpP protease for degradation (22, 23). Thus, some AAA+ proteins have been shown to use a helicase-like mechanism for protein remodeling. Both NSF and ClpX recognize their protein substrates through interactions in the central pore; a conserved sequence motif (YVG motif), located on a loop in a hydrophobic patch of the NSF-D1 pore, is required for SNARE complex binding (6). This motif is highly conserved in other AAA+ proteins that use a threading mechanism and is only present on the active AAA+ domain (17). In ClpX, this loop undergoes nucleotide-dependent conformational changes, and mutations drastically decrease the efficiency of the motor, suggesting that this loop is directly involved in substrate polypeptide translocation (24, 25).

To investigate the mechanism by which NSF disassembles SNAREs and to gain insight into the motor properties of this molecular machine, we developed a real-time method to measure SNARE disassembly. We find that NSF-driven disassembly of the SNARE complex takes ∼5 s and uses ∼50 ATP. This coupling ratio of ∼50 ATP per SNARE is insensitive to changes in salt conditions despite large salt effects on both the ATPase rate and the disassembly rate. NSF can disassemble SNAREs that are twice the normal length, suggesting a processive mechanism. Single-turnover burst measurements show that one NSF binding event can be sufficient for SNARE disassembly. We propose a model for NSF action based on a threading/helicase-like mechanism and speculate that it is driven by sequential ATP hydrolysis in the hexamer.

## EXPERIMENTAL PROCEDURES

### 

#### 

##### Protein Production

All proteins were expressed in BL21 (DE3) at 37 °C using autoinducing LB medium (26). Supplemental Fig. S1 shows the amino acid sequences of all expressed proteins. All protein purification steps were performed at 4 °C. Cell pellets from 4 liters of culture were suspended in 200 ml of 50 mm NaP_i_, pH 8, 300 mm NaCl, 20 mm imidazole, and 0.5 mm TCEP (Ni-NTA buffer) (except for NSF where Tris was substituted for NaP_i_) supplemented with 1 mm PMSF and Complete EDTA-free protease inhibitor mixture tablets (Roche Applied Science). Cells were lysed by two passages through a M-110-EH Microfluidizer (Microfluidics) at 15,000 p.s.i. The lysate was clarified by initial centrifugation at 10,000 rpm in a Beckman JA-20 rotor for 10 min followed by centrifugation at 40,000 rpm in a Beckman Ti45 rotor for 1.5 h.

Chinese hamster NSF with a noncleavable C-terminal His tag was expressed from pET28a. After lysis and clarification, the lysate was loaded onto a 5-ml Ni-NTA agarose column (Qiagen), washed with 100 ml of 50 mm Tris, pH 8, 300 mm NaCl, 20 mm imidazole, and eluted in the wash buffer supplemented with 350 mm imidazole. Fractions containing NSF were pooled and supplemented with 1 mm MgCl_2_, 1 mm ATP, and 10% glycerol before being concentrated using an Amicon Ultra-15 centrifugal filter with 30-kDa molecular mass cut off (Millipore) to reduce the volume to 2 ml. The protein was then run through a Superdex 200 16/600 column (GE Healthcare) that was equilibrated with 50 mm Tris, pH 8, 100 mm NaCl, 10% glycerol, 1 mm MgCl_2_, 1 mm ATP, and 1 mm DTT.

Soluble neuronal SNARE complex was produced by co-expressing wild type SNAP-25A with syntaxin-1A_1–265_ S249C K253C and hexa-His-tagged synaptobrevin-2_1–96_ using the Duet expression system (Novagen). The clarified lysate was loaded onto a 3-ml Ni-NTA agarose column, washed with 100 ml of Ni-NTA buffer containing 7.5 m urea, and then washed with 100 ml of Ni-NTA buffer. If the complex was to be unlabeled, it was then eluted with Ni-NTA buffer containing 350 mm imidazole. If the protein was to be labeled, the resin was washed with 20 ml of PBS buffer containing 0.5 mm TCEP followed by 20 ml of thoroughly degassed PBS. After draining excess PBS out of the column, the resin was suspended in 5 ml of degassed PBS, and 1 mg of Oregon Green 488 maleimide (Invitrogen) (dissolved in 400 μl of anhydrous dimethyl sulfoxide) was added. The column was capped and gently mixed by rotation for 30 min at 4 °C. The column was then drained, and the resin was washed with 100 ml of PBS. The protein was then eluted with Ni-NTA buffer supplemented with 350 mm imidazole. After Ni-NTA chromatography, the SNARE complexes were cleaved by overnight digestion with tobacco etch virus protease at 4 °C and subjected to size exclusion chromatography using a Superdex 200 10/300 column (GE Healthcare) that was equilibrated with 50 mm Tris-HCl, pH 8, 100 mm NaCl, 0.1 mm TCEP (SNARE buffer). The complex showed a single symmetric peak when subjected to size exclusion chromatography. The complex could be completely disassembled by NSF as judged by the complete loss of SDS-stable SNARE complex on SDS-PAGE gels.

Both the “extended SNARE” (Ext-SNARE) complex and the mini-SNARE complex (1) were purified using the same procedure as the soluble SNARE complex, with the exception that the mini-SNAREs were subjected to an additional ion exchange chromatography step using a Mono-Q 5/50 column (GE Healthcare). The pooled fractions were then dialyzed into SNARE buffer.

The GCN4 tetramer was expressed from pET28a as a duplication of the GCN4 pLI mutant (27) with an N-terminal hexahistidine and maltose-binding protein tag. The Ni-NTA eluate was digested with tobacco etch virus protease while being dialyzed overnight into SNARE buffer. Uncleaved protein was removed by passing the sample through an amylose affinity column (New England Biolabs), and the protein was further purified by size exclusion chromatography on a Superdex 200 10/300 column (GE Healthcare). The pooled protein fractions were then subjected to ion exchange chromatography on a Mono-Q 5/50 column with a gradient of 50–500 mm NaCl over 80 column volumes in buffer containing 50 mm Tris-HCl, pH 8, 0.1 mm TCEP. The pooled fractions were then dialyzed into SNARE buffer.

Rat α-SNAP was expressed with an N-terminal tobacco etch virus cleavable decahistidine tag from a codon-optimized plasmid using the pJexpress414 backbone (DNA 2.0). The clarified lysate was loaded onto a 5-ml Ni-NTA agarose column, washed with 200 ml of Ni-NTA buffer, and eluted using 500 mm imidazole in Ni-NTA buffer. Tobacco etch virus protease was added to pooled fractions, and the sample was dialyzed overnight into 50 mm Tris-HCl, pH 8, 50 mm NaCl, and 0.5 mm TCEP (α-SNAP buffer) at 4 °C. The protein was then subjected to anion exchange chromatography using a Mono-Q HR 10/100 column (GE Healthcare) and eluted with a gradient of 0–500 mm NaCl in α-SNAP buffer. Fractions containing protein were pooled, concentrated to 3 ml using a 10-kDa molecular mass cutoff Amicon Ultra-15 centrifugal filer (Millipore), and subjected to size exclusion chromatography using a Superdex 200 16/600 column that was equilibrated with α-SNAP buffer.

**FIGURE 2.**
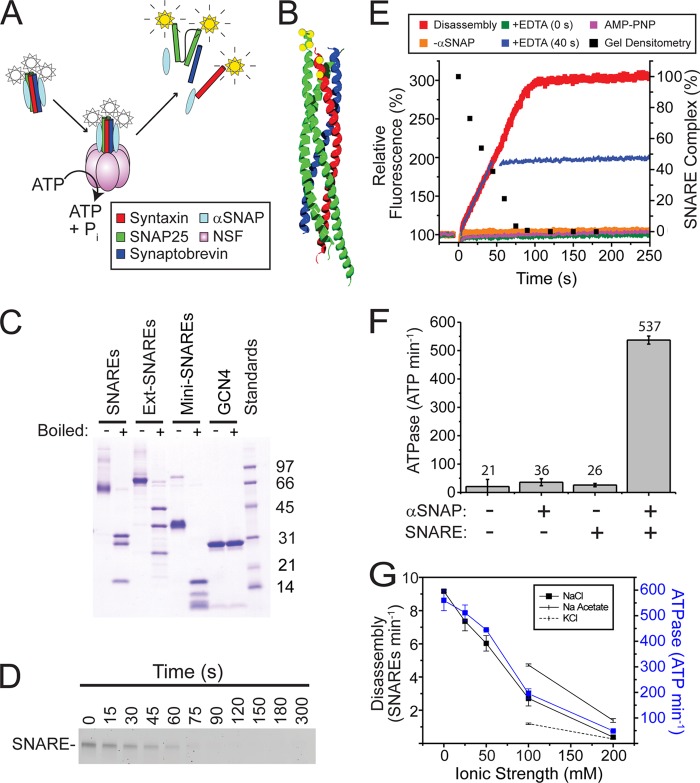
**ATP-powered disassembly of SNAREs by NSF.** *A*, schematic representation of bulk fluorescence-dequenching-based SNARE disassembly assay. *Stars* represent the fluorescent dye, Oregon Green 488. *B*, structure of the minimal neuronal SNARE complex (Protein Data Bank (PDB) 1N7S) illustrating sites used for labeling with Oregon Green maleimide for the dequenching assay. Helices are colored using the scheme in *panel A. Yellow spheres* represent the cysteine residues used for labeling. *C*, SDS-PAGE gel of purified proteins used in this study. Proteins were suspended to 0.5 μg/μl in Laemmli sample buffer with DTT and either boiled (+) or not (−). 10 μl were loaded into each lane of an Any kD denaturing polyacrylamide gel (Bio-Rad). *D*, SDS-PAGE-based SNARE disassembly assay. *E*, fluorescence dequenching-based disassembly assay (*colored lines*, *left axis*). The *black arrow* indicates the addition of NSF at time 0. *Red*, typical disassembly assay. *Yellow*, same assay performed in the absence of α-SNAP. *Green*, disassembly assay performed in the presence of 10 mm EDTA. The *blue curve* shows a disassembly assay that was stopped at 40 s by the addition of 10 mm EDTA. For comparison, densitometry was performed on the gel in *panel C* and overlaid (black *squares*, *right axis*). *F*, SNARE-dependent activation of NSF ATPase activity. Data shown are the average of three independent assays ± S.E. collected at 37 °C. *Numbers* shown above each bar represent the average rates. *G*, effect of ionic strength on ATP-driven SNARE disassembly by NSF. *Black squares* and *blue squares*, the effect of added NaCl on the disassembly rate (*black squares*) and SNARE-activated ATPase rate (*blue squares*) of NSF. For comparison, the effects of 100 and 200 mm KCl (*solid line*) and NaCH_3_COO (*dashed line*) on the disassembly rates are shown. All data shown are the average of triplicate assays with standard errors.

The purified SNARE and GCN4 proteins are shown in Fig. 2*C*. Immediately after purification, all proteins were aliquoted, flash-frozen in liquid nitrogen, and stored at −80 °C. After thawing, aliquots were stored on ice and used the same day. All protein concentrations were estimated using the Bradford method (28).

##### Fluorescence Dequenching-based SNARE Disassembly Assay

Disassembly assays were performed at 37 °C in a Hitachi F-4500 spectrofluorometer equipped with a water-jacketed cuvette holder. Standard assays were performed in 240-μl volumes containing 25 mm Tris-HCl, pH 8, 2 mm ATP, 4 mm MgCl_2_, 2 mm β-mercaptoethanol, 3 mm phosphoenolpyruvate, 0.3 mm NADH, 12.5 units/ml pyruvate kinase, 18.75 units/ml lactate dehydrogenase, 2.2 μm α-SNAP, and 442 nm Oregon Green-labeled SNARE. The cuvette was placed in the spectrofluorometer, and the temperature was equilibrated before the reaction was started by adding 24 nm NSF (hexamer concentration). Time series were collected for 5 min with excitation collected at 490 nm and emission collected at 530 nm. Each trace was normalized to the final plateau value of the fluorescence (averaged over the last 20 s and corresponding to 100% disassembly), and a short initial region of each trace (covering <50% of the total fluorescence change) was fit to a line to determine the initial velocity.

##### ATP Hydrolysis Assay

Coupled ATPase reactions were conducted under the identical conditions to the disassembly assays, except that SNAREs were omitted from the initial incubation. The reaction was set up, and the temperature was equilibrated to 37 °C using the cuvette holder on a SpectraMax Plus plate reader (Molecular Devices). The reaction progress was followed by monitoring the absorbance at 340 nm. After the NSF-only base line was established, the activated activity was measured by the addition of unlabeled SNAREs. The hydrolysis rates were determined from the initial slope (within the first 30 s) of the resulting *A*_340_ *versus* time graph.

##### Single-turnover Kinetic Burst assay

Single-turnover burst assays were performed by premixing 750 nm Oregon Green-labeled SNARE, 1126 nm NSF hexamer, and 3750 nm α-SNAP in 800 μl of buffer containing 25 mm Tris-HCl, pH 8, 2 mm ATP, 2 mm EDTA, and 1 mm β-mercaptoethanol and incubating at 37 °C for 5 min. The dark trap was then added in a volume of 700 μl containing 2.3 μm unlabeled SNARE and 11.7 μm α-SNAP in buffer consisting of 50 mm Tris-HCl, pH 8, 2 mm ATP, and 1 mm β-mercaptoethanol. The mixture was placed in a 2-ml quartz fluorescence cuvette with a magnetic stir bar and placed in the jacketed cuvette holder of the spectrofluorometer at 37 °C. Disassembly was started by the addition of MgCl_2_ to 5 mm, and disassembly was measured as described above. The burst magnitude was estimated by visually identifying the point at which the slope of the fluorescence trace abruptly changes at the end of the initial fluorescence jump. The fluorescence value at this point was normalized to the final fluorescence plateau for each trace.

## RESULTS

### 

#### 

##### Development of a Bulk NSF Activity Assay

To gain insight into the mechanism of NSF-driven SNARE disassembly, routine quantitative disassembly assays are needed. Building on previous real-time assays based on FRET between fluorescent proteins (29), we developed an assay based on fluorescence dequenching. In addition to bulk disassembly rates, the assay allows for estimation of the ATPase coupling ratio and for single-turnover burst analysis of disassembly. Fig. 2*A* shows the general scheme for a bulk fluorescence dequenching-based disassembly assay using SNARE complex devoid of the transmembrane domains on syntaxin and synaptobrevin. Neuronal SNAREs expressed without the transmembrane domains form an SDS-resistant soluble complex that can be disassembled by NSF (30). We engineered a variant of this soluble SNARE complex with six cysteine residues (the four endogenous residues on SNAP-25 and the two engineered syntaxin S249C/K253C residues) in close proximity to each other (Fig. 2*B*). SNAREs were co-expressed using the Duet expression system (Novagen), purified, and labeled with the self-quenching dye Oregon Green 488 maleimide (Invitrogen). The purity of the purified SNARE complex is high (Fig. 2*C*, *left two lanes*). In our hands, coexpression results in SNARE complexes that appear substantially more monodisperse than SNAREs assembled *in vitro* from the constituent proteins (see “Experimental Procedures”).

Upon the addition of α-SNAP, NSF, and Mg-ATP, the labeled complex was disassembled as judged by loss of SDS-resistant complex in SDS-PAGE experiments (Fig. 2*D*). This disassembly was also monitored in real time by measuring the fluorescence increase of the Oregon Green dye at 530 nm (Fig. 2*E*). The fluorescence initially increased linearly, reaching a plateau with approximately the same rate at which SNARE complex completely disappears in the SDS-PAGE monitored assay (Fig. 2, *D* and *E*). The fluorescence trace was used to determine the initial velocity of the reaction under multiple turnover conditions. At 37 °C one NSF hexamer disassembles 10.7 ± 0.2 SNARE complexes per minute. This corresponds to 5.6 ± 0.1 s for a single NSF hexamer to disassemble a single SNARE complex.

The fluorescence change observed in our disassembly reaction was dependent on ATP, α-SNAP, NSF, and Mg^2+^ as expected and did not occur in the presence of AMP-PNP (Fig. 2*E*). The addition of EDTA completely abolished disassembly activity. The addition of EDTA in the middle of the reaction caused the fluorescence to immediately stabilize and remain constant, showing that SNARE complex reassembly does not occur on the timescale of assay (Fig. 2*E*). The fluorescence dequenching assay may thus be used to directly measure the rate of NSF-driven SNARE disassembly.

##### SNARE-activated ATPase Activity

To correlate the SNARE disassembly rate with the rate of ATP hydrolysis, we performed a coupled ATPase reaction under identical conditions to the disassembly reaction (Fig. 2*F*). Unlike previous studies that showed only moderate SNARE-induced stimulation (31, 32), we noticed that SNAREs significantly activated the ATPase activity of NSF when KCl and NaCl were omitted (Fig. 2*F*). Activation was dependent on α-SNAP. The activation was 26-fold for the data shown in Fig. 2*F* and varied from 26- to 31-fold depending on the preparation. In the presence of excess SNAREs and α-SNAP, one NSF hexamer hydrolyzes 537 ± 14 ATP min^−1^.

##### Coupling Ratio for NSF-driven SNARE Disassembly

Because the ATPase activity was measured under the same conditions as the disassembly rate and we measure the SNARE-activated ATPase activity (the basal ATPase rate was negligible: ∼4% of the activated rate), we can calculate a coupling ratio for NSF in terms of the number of ATP molecules hydrolyzed during the disassembly of a single SNARE. Based on the measurements above, in saturating concentrations of Mg-ATP, SNARE and α-SNAP, we measure a coupling ratio of 50 ± 2 ATP per SNARE disassembled.

##### Effect of Ionic Strength on Coupling Ratio

To investigate how tightly the ATPase activity of NSF is coupled to NSF disassembly, we examined the robustness of the coupling ratio to perturbations of the disassembly reaction. We initially noticed that high ionic strength conditions inhibit NSF-driven SNARE disassembly, so we investigated how ionic strength affected the coupling ratio. As shown in Fig. 2*G*, NaCl strongly inhibits both the NSF-driven disassembly rates and the SNARE-activated ATPase rates. These two activities are well correlated, showing that NaCl has little effect on the coupling ratio, but instead inhibits the whole system.

##### Structural Features of SNAREs Required for Recognition and Disassembly by NSF and α-SNAP

**FIGURE 3.**
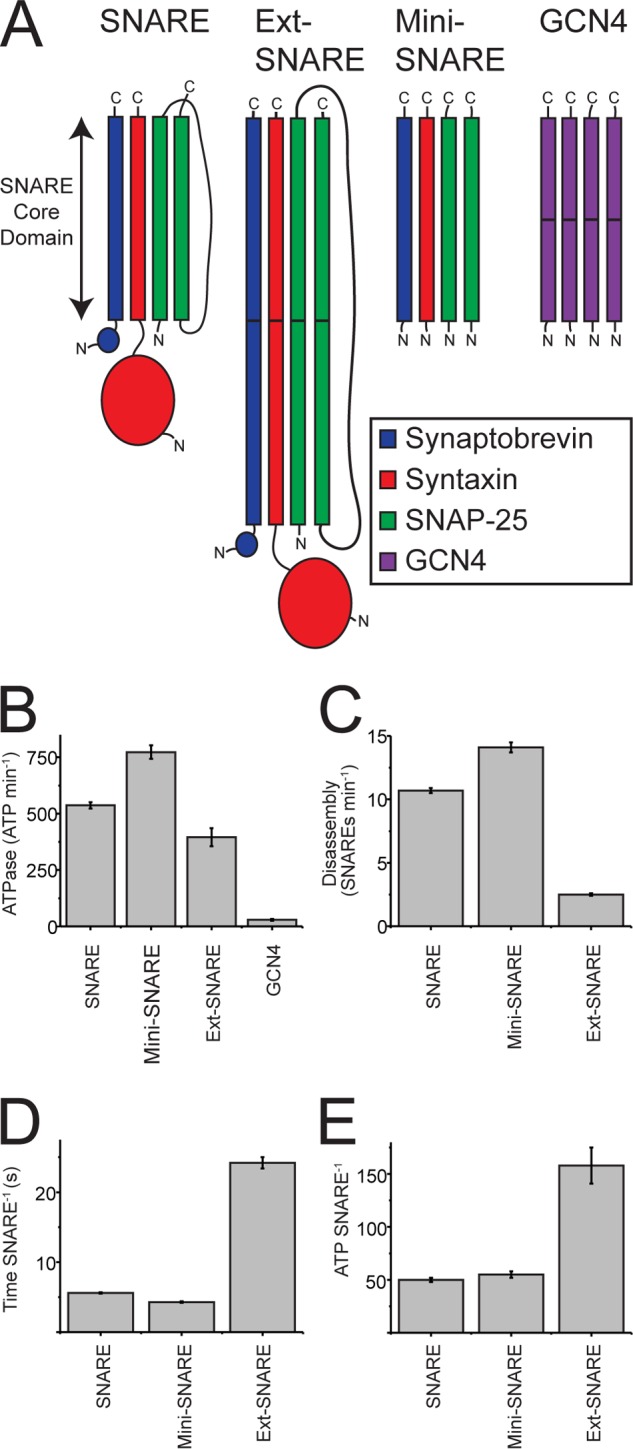
**NSF-driven disassembly of engineered SNARE substrates.** *A*, schematic diagram illustrating the domain structure of the different SNARE substrates used in this study. *B*, SNARE-activated ATPase activity. *C*, NSF-driven disassembly. *D*, average time required for one NSF hexamer to disassemble a single SNARE complex. *E*, number of ATP molecules hydrolyzed for each SNARE molecule disassembled. Data shown are the average of three independent assays ± S.E. collected at 37 °C.

To learn about the mechanism of NSF-driven SNARE disassembly, we assessed the structural features of the SNARE complex required for NSF and α-SNAP to recognize and disassemble it. In addition to the soluble SNARE complex, we also co-expressed a “minimal” soluble SNARE complex (1) consisting of only the SNARE core domains (*i.e.* missing the Habc domain of syntaxin and the connecting loop of SNAP-25). Separately, we purified an unrelated four-stranded coiled-coil: a tandem repeat of the GCN4-pLI tetramer (27) and an Ext-SNARE complex where the SNARE domains were duplicated (Fig. 3*A*). All constructs were SDS-stable, including the GCN4 tetramer, which was stable in SDS even after boiling the sample (Fig. 2*C*).

The minimal SNARE complex was disassembled by NSF (Fig. 3, *C* and *D*), with slightly higher velocities than the full-length soluble construct (14.1 ± 0.4 SNARE NSF^−1^ min^−1^, or 4.3 ± 0.1 s per SNARE when compared with 5.6 ± 0.1 s per SNARE for the full-length soluble construct). This demonstrates that regions outside the core of the SNARE complex are not required to be recognized and acted on by NSF and/or α-SNAP. The elevated rate of disassembly was correlated with an increased rate of ATP hydrolysis (Fig. 3*B*), yielding a similar coupling ratio of 55 ± 3 ATP per SNARE complex (Fig. 3*E*). Although these two SNARE complexes require the same amount of ATP molecules to disassemble them, the slower rate of disassembly shown by the full-length soluble SNAREs suggests that additional protein domains can impede the rate of disassembly.

To probe whether NSF and α-SNAP recognize any specific features of the SNARE complex beyond the overall parallel four-helix bundle architecture, we expressed a nonrelated, engineered four-helix bundle, derived from the GCN4 coiled-coil domain (27). To make the GCN4 tetramer a comparable length to a SNARE coil, it was expressed as an in-frame duplication and resulted in an SDS-stable tetramer as judged by SDS-PAGE (Fig. 2*C*). The GCN4 tetramer did not activate the ATP hydrolysis rate of NSF (Fig. 3*B*). This result suggests that specific features of the SNARE complex, beyond the four-stranded parallel helical bundle architecture, are recognized by NSF and α-SNAP.

To challenge the global unwinding model for NSF-driven SNARE disassembly, we created a SNARE complex in which the SNARE domains were doubled in length (referred to as the Ext-SNAREs). Disassembly of such a construct would be difficult to reconcile with a global unwinding model because NSF would need to act globally on a complex that is now twice the size of its usual substrate. However, processive unwinding models predict that Ext-SNARE should be disassembled because unwinding may progress sequentially through the initial and extended portions of the helical bundle. Although the Ext-SNARE complex required more ATP (158 ± 17 ATP Ext-SNARE^−1^) and was disassembled at a much slower rate (2.5 ± 0.1 Ext-SNARE NSF^−1^ min^−1^ or 24.2 ± 0.8 s per Ext-SNARE), it was disassembled by NSF, suggesting that NSF processively unwinds the complex.

##### Single-turnover Burst Kinetics

To distinguish between distributive and processive models for SNARE disassembly, we used a single-turnover approach, analogous to experiments performed on DNA helicases (33–35) and used to measure helicase processivity (a quantity that is often reported as the average number of bases unwound per encounter). In these single-turnover helicase experiments, labeled DNA is unwound in the presence of an unlabeled nucleic acid “trap.” The trap acts to sequester the enzyme if it falls off the DNA substrate before it reaches the end of the molecule. The probability that the helicase will completely unwind a given length of DNA substrate (which can be measured as the fraction of substrate molecules that are completely unwound during the single-turnover assay) is directly related to the processivity of the enzyme.

**FIGURE 4.**
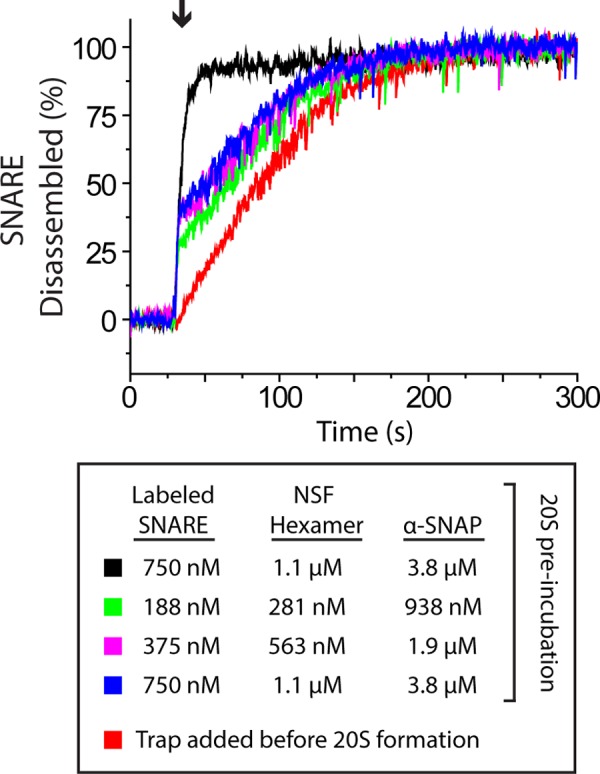
**Single-turnover burst kinetics of NSF-driven SNARE disassembly.** 20S complex was formed by premixing Oregon Green 488-labeled SNARE complex with a 1.5-fold excess of NSF hexamer and 5-fold excess of α-SNAP in the presence of ATP and 1 mm EDTA. A 50-fold excess of unlabeled SNARE was added as a trap, either before or after the initial 20S complex formation as indicated. At the time indicated by the *arrow*, the disassembly reaction was started by the addition of MgCl_2_ to 5 mm. *Black*, no trap added; *red*, trap added before NSF; *green*, *purple*, and *blue*, trap added after 20S complex formation. 20S complex was formed by incubation at 37 °C for 5 min with 188 nm labeled SNARE, 281 nm NSF hexamer, and 938 nm α-SNAP (*green*); 375 nm labeled SNARE, 563 nm NSF hexamer, and 1875 nm α-SNAP (*purple*); or 750 nm labeled SNARE, 1.1 μm NSF hexamer, and 3.8 μm α-SNAP (*blue*).

For single-turnover NSF assays, we preincubated labeled SNARE complex with a 1.5-fold molar access of NSF and a 5-fold molar excess of α-SNAP in the presence of ATP and EDTA. Under these conditions, we expect all of the labeled SNAREs should form the 20S complex with NSF and α-SNAP.^4^ The samples were then diluted into buffer containing a 50-fold excess of unlabeled SNAREs (dark trap), and the reaction was started by the addition of excess Mg^2+^. If a single NSF can disassemble a SNARE complex without dissociation and rebinding to another SNARE complex, we expect to see a rapid burst in the fluorescent signal. If NSF is infinitely processive, then we expect this burst to go to completion as all the SNARES should be disassembled during the burst phase. If NSF is moderately processive, then only a population of the 20S complexes will be disassembled during the burst. In this case, the burst magnitude will be proportional to the fraction of molecules disassembled and tells us the probability that a single NSF binding event results in disassembly. The NSF that dissociates is 50 times more likely to bind an unlabeled SNARE and contributes only to an overall upward creep that is easily distinguishable from the burst magnitude (Fig. 4, *red line*).

Single-turnover NSF assays show a significant burst phase. In the experiments shown in Fig. 4, a burst size of ∼40% is seen. After the burst, the rate immediately slowed down to a rate similar to the non-preincubated control. A burst size of ∼40% suggests a processive enzyme with a probability of finishing a single round of disassembly before falling off the SNARE complex of ∼0.4. This probability may be an underestimation due to incomplete 20S formation or the presence of inactive NSF. We performed preincubations at varying concentrations of 20S components; a trace collected at the lowest protein concentration (Fig. 4, *green line*) shows a reduced burst magnitude, which may be due to incomplete complex formation. All traces show substantial processivity for NSF and are consistent with a processive unwinding model.

##### NSF Shows Substrate Inhibition at High Concentrations of ATP

**FIGURE 5.**
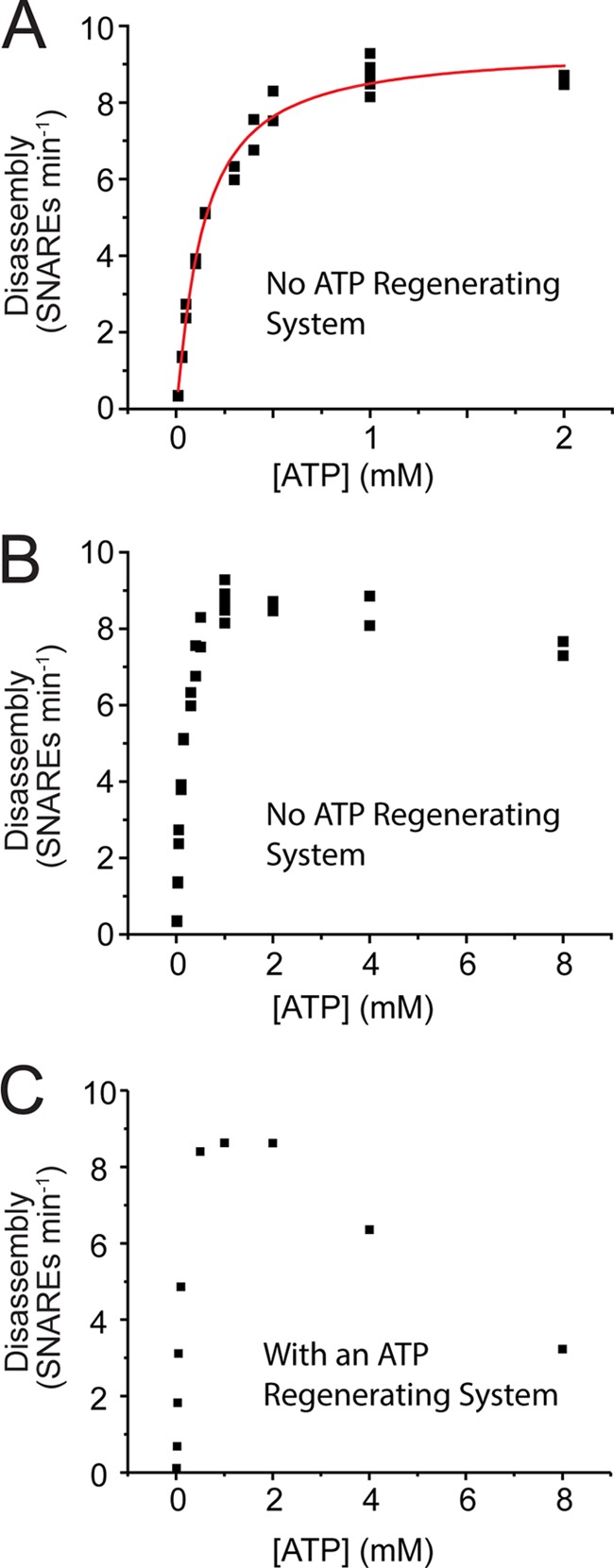
**Kinetic analysis of ATP-driven SNARE disassembly by NSF.** Disassembly assays were set up and measured as described under “Experimental Procedures,” except that [ATP] was varied and MgCl_2_ was added to a concentration that was 1 mm above [ATP] in each case. *A*, Hill analysis with no ATP-regenerating system. Data were fit to a Hill equation yielding *K*_0.5_ = 0.14, *K*_cat_ = 9.4 SNAREs min^−1^, *n* = 1.12 with an *R*^2^ of 0.984. *B* and *C*, kinetics including high [ATP] conditions, showing inhibition at high [ATP]. *B*, no ATP-regenerating system (data from *panel A* are replotted along with higher [ATP] data). *C*, ATP-regenerating system added.

Most previous studies of ATP hydrolysis by NSF have been performed with the uncoupled basal ATPase (32). We exploited our observation of strong SNARE activation of ATPase to study the kinetics of SNARE-coupled ATP hydrolysis. We measured the initial SNARE disassembly rates as a function of ATP concentration (Fig. 5). At ATP concentrations under 2 mm, NSF is well fit by a Hill equation, with an apparent *K*_0.5_ of 0.14 mm, a *K*_cat_ of 9.4 SNARES min^−1^, and a Hill coefficient *n* = 1.1. At ATP concentrations above 2 mm, NSF showed substrate inhibition. Substrate inhibition has been seen for other ring ATPases (36) but is shown for NSF for the first time here. Substrate inhibition was seen with or without an ATP-regenerating system, but was much more pronounced in the presence of ATP regeneration (compare Fig. 5, *B* and *C*).

## DISCUSSION

**FIGURE 6.**
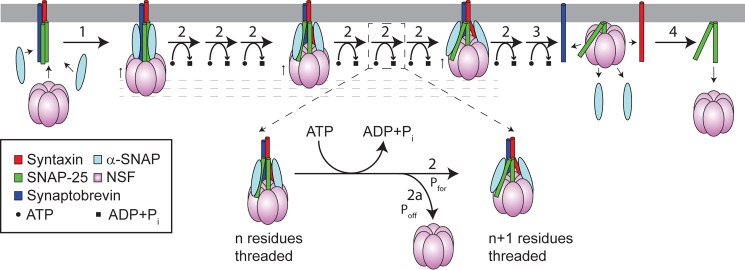
**Model for NSF-driven SNARE disassembly.** For simplicity, the model shows only the core SNARE helices (and their respective transmembrane domains), which are drawn as *cylinders*. Binding of α-SNAP to the fully assembled SNARE complex primes it for binding of NSF (*step 1*). Sequential hydrolysis of ATP in the NSF-D1 hexamer results in sequential conformational changes that drive the threading of the N terminus of SNAP-25 into the central pore (*steps 2*). In one step (shown enlarged), a single residue is translocated, coupled to hydrolysis of one ATP. Complete disassembly could take up to 60 steps; for simplicity, only three intermediate steps are shown. NSF is not obligately processive and can dissociate at any point (*step 2a*). After the unwinding progresses beyond a critical nucleus, the SNARE core complex is destabilized (*step 3*). This results in the complete disassembly of the SNARE complex, and α-SNAP, syntaxin, and synaptobrevin are free to diffuse away. The final step is dissociation of NSF from SNAP-25 (*step 4*).

A defining feature of the AAA+ superfamily of proteins is a structurally and functionally homologous core AAA+ domain (21). Our data support a mechanism for NSF that is reminiscent of a number of AAA+ motors that translocate diverse substrates (37); this group includes AAA+ helicases and ATP-fueled proteases (ClpXP, HslUV, Lon, FtsH, and the 26 S proteasome) (38). Like these homologous AAA+ proteins, NSF contains the conserved YVG motif on pore loop one of NSF-D1 (17, 21). This motif, present only on the active AAA+ domain of translocating proteins, is involved directly in substrate binding and suggested to play a critical role in translocation (39). Consistent with the proposal that NSF is also a peptide translocase, this motif is essential to the function of NSF (6). We propose that NSF utilizes a sequential ATP hydrolysis mechanism that is coupled to sequential conformational changes that drive a threading mechanism (Fig. 6).

Two of our results suggest that NSF works by a processive unwinding mechanism. First, the large burst phase under single-turnover conditions (Fig. 4) rules out a distributive model for the mechanism of NSF. Second, NSF can disassemble the double length Ext-SNARE complex. This fact is hard to reconcile with a global unwinding model such as the socket-wrench model. In a processive unwinding model, NSF can continue to work on the Ext-SNARE proteins until the complex disassembles.

Based on our experimental support for processive unwinding and on the homology and similarities between NSF and other known AAA+ threading translocases, we propose that NSF also uses a translocation mechanism. The model shown in Fig. 6 explains our results, is consistent with the data in the literature, and forms our working hypothesis for future experiments. First α-SNAP recognizes and binds to the SNARE core domain. Because the minimal SNARE complex and not the GCN4 tetramer is recognized by NSF and α-SNAP (Fig. 3), there must be some sequence-specific contact with the surface of the SNARE core complex. In a mechanism homologous to ClpX, sequential ATP hydrolysis on NSF-D1 pulls on one of the SNARE proteins, translocating it into the pore of the hexamer (*steps 2* in Fig. 6). The repetitive hydrolysis of ATP drives the sequential threading of the protein through the pore of the hexamer (repeating *step 2* in Fig. 6; for each step, there is one ATP molecule hydrolyzed). During translocation, NSF can occasionally fall off the substrate (*step 2a*).

Recent low resolution electron microscopy studies show that the central pore in the NSF hexamer is ∼7 Å at its smallest diameter (9), just big enough to feed through an unfolded polypeptide. SNAP-25 may be the most reasonable candidate to be threaded into the NSF pore because syntaxin and synaptobrevin homologues often have very large N-terminal domains, although it remains possible that, as with other AAA+ unfoldases (40–42), threading could initiate from an internal loop. The fluorescence intensity change seen during NSF-driven disassembly of our labeled SNARE complex also suggests that synaptobrevin is not translocated. Because syntaxin and SNAP-25 form a stable “binary complex” (43), and synaptobrevin does not have any fluorescent signal (it is cysteine-free and unlabeled), the increase in the fluorescence signal during our assay indicates disassembly of the binary complex, which would not be obligatory if synaptobrevin were translocated into NSF.

A criticism of the threading/helicase-like model for SNARE complex disassembly by NSF has been that the membrane poses a steric constraint, disallowing the protein to be threaded entirely through the pore and thus requiring a mechanism for NSF removal from the threaded protein (21). Our single-turnover measurements are consistent with a model in which NSF is highly but not obligatorily processive and occasionally slips off the SNARE complex substrate. This provides an inherent mechanism for release of NSF once disassembly is complete (*step 4* in Fig. 6). Another speculative feature of this model is that it may provide an inherent mechanism to prevent reassembly of the SNAREs subunits immediately following disassembly; NSF protects the threaded protein from reassembly, giving the other subunits a chance to diffuse away.

In the threading mechanism we propose, one ATP molecule is hydrolyzed per unit or step of translocation. ClpX hydrolyzes ∼1 ATP molecule per residue of substrate peptide translocated (44). Our measurement of 50 ATP per SNARE disassembly event is similar to the number of residues in one helix of the four-stranded helical bundle (∼60 residues). Two effects should be noted. 1) This number of ATP molecules includes ATPs wasted in unproductive binding and unwinding events (Fig. 4), which may constitute as much as 60% of NSF-SNARE encounters; this leads to an *overestimate* of the number of ATP molecules hydrolyzed per residue translocated. 2) Although the SNARE domains are each ∼60 residues in length, it is likely not necessary to thread all 60 residues into the NSF pore (nor might it be possible to do so *in vivo*, given the geometric constraints of the membrane). Threading need only proceed to a critical point where the remaining helical bundle structure becomes unstable and the SNAREs come apart (*step 3* in Fig. 6); this leads to an *underestimation* of the number of ATP molecules hydrolyzed per residue translocated. The fundamental step size of the putative unwinding mechanism can therefore not be directly calculated from our measurements, but our results are nonetheless consistent with models in which ∼1 residue is translocated per ATP hydrolyzed.

Under physiological conditions, the total free energy released by hydrolyzing 50 ATP during SNARE unwinding by NSF is ∼1000 *k_B_T*. This is substantially greater than the free energy change of 65 *k_B_T* measured for the unfolding of SNAREs using optical tweezers (45). Similarly, many helicases have been shown to hydrolyze ∼1 ATP per bp unwound (20, 37, 46–48), consuming substantially more free energy (∼20 *k_B_T*/bp) than is required for strand separation (∼2 *k_B_T*/bp) (49). Our data suggest an iterative stepwise mechanism in which ATP hydrolysis events are coupled to small changes, for instance translocation of one unit in a threading translocase. Attempts to disrupt NSF-driven SNARE disassembly with conditions of high ionic strength (Fig. 2*G*) or by removing large protein domains (Fig. 3) have no effect on the coupling ratio. Indeed, even doubling the length of the SNARE core domain only results in an increase in the coupling ratio by ∼2–3-fold (Fig. 3*B*-E), which can be quantitatively explained by futile encounter events due to limited processivity.

We present here a novel real-time assay for measuring NSF-driven SNARE disassembly kinetics and a method to produce soluble SNARES through co-expression. Our assay allows for the estimation of the coupling ratio of NSF by measuring ATP hydrolysis rates under the same conditions as disassembly. At 37 °C one NSF hexamer takes 5.6 ± 0.1 s to disassemble a single SNARE complex. This is in reasonable agreement with previously published results that show NSF taking between 2 and 4 s to disassemble a SNARE (29, 50).

Using our new disassembly assay, we were able to measure the NSF-driven disassembly of a minimal SNARE complex (Fig. 3). The SNARE core domain, which is the minimal sequence required to form a stable SNARE complex (1, 51), is sufficient for recognition and disassembly by NSF and α-SNAP. However, an unrelated engineered GCN4 tetramer that forms a four-helix bundle does not activate the ATPase activity of NSF, suggesting that there must be some sequence-specific contacts between SNAREs and either NSF or α-SNAP.

A highly conserved feature of the SNARE complex is the “zero layer” of ionic residues that reside in the interior of the SNARE hydrophobic core (1, 51, 52). These residues have been proposed to ensure that the strands of the four-helix bundle assemble in register (1, 51) and have been described as a halfway point for SNARE complex zippering (45, 53). The zero-layer has also been proposed to play a key role in NSF-driven disassembly (54), but recent work has shown that disassembly can still occur when these charged residues are mutated (29). In the context of our model, the latter result may reflect the robustness of the threading mechanism; once unwinding has been initiated, it may proceed regardless of the details of the interactions in the core. We also show here that the SNARE complex activates the ATPase activity of NSF (Fig. 2*F*). A previous study suggested that SNARE complex does not significantly stimulate the ATPase activity of NSF (32), although α-SNAP can stimulate the activity by up to 2.5-fold (55). In marked contrast, here we show a very large (26-fold) activation of the ATPase rate that is both SNARE-specific and α-SNAP-specific, in agreement with other recent results (6). Neither SNAREs nor α-SNAP alone significantly stimulate NSF, but the combination of the two results in a significant activation (Fig. 2*F*). This activation has probably not been detected in past experiments because of the high ionic strength that was typically used in ATPase assays. High salt concentrations inhibit both the ATP hydrolysis and the SNARE disassembly rates of NSF (Fig. 2*G*). The degree of salt inhibition depends on which salt used (Fig. 2*G*) and may be less important *in vivo*, where localization at the membrane and macromolecular crowding can compensate for weakened interactions between NSF, α-SNAP, and SNAREs.

Taken together our results are consistent with a processive model for the mechanism of NSF-driven SNARE disassembly. Coordinated ATP hydrolysis on the NSF hexamer drives a processive threading type of mechanism in which one of the SNARE proteins is threaded into the pore of the NSF hexamer. This model may be further tested using single molecule approaches.

## Supplementary Material

Supplemental Data

## Footnotes

4 Vivona, A., Cipriano, D. J., O‘Leary, S., Li, Y. H., Fenn, T. D., and Brunger, A. T. (2013) Disassembly of all SNARE complexes by *N*-ethylmaleimide-sensitive factor (NSF) is initiated by a conserved 1:1 interaction between α-soluble NSF attachment protein (SNAP) and SNARE complex. *J. Biol. Chem.* **288,** 10.1074/jbc.M113.489807.

## Footnotes

3 The abbreviations used are:

AAA+ ATPases: ATPases associated with various cellular activities
α-SNAP: α-soluble NSF attachment protein
Ni-NTA: nickel-nitrilotriacetic acid
NSF: *N*-ethylmaleimide-sensitive factor
Ext-SNARE: extended SNARE
TCEP: tris(2-carboxyethyl)phosphine
AMP-PNP: adenosine 5′-(β,γ-imino)triphosphate.
