# Supplementary material for: Processive ATP-driven Substrate Disassembly by the N-Ethylmaleimide-sensitive Factor (NSF) Molecular Machine
Source: J Biol Chem. 2013 Jun 17;288(32):23436–45. doi: 10.1074/jbc.M113.476705 (PMC4520572; doi:10.1074/jbc.M113.476705)
Supplement: Supplemental Data [file supp_288_32_23436__index.html]

Processive ATP-driven disassembly of SNARE complexes by the N-ethylmaleimide sensitive factor molecular machine — Processive ATP-driven Substrate Disassembly by the N-Ethylmaleimide-sensitive Factor (NSF) Molecular Machine — NSF-driven SNARE Disassembly — Supplemental Data 

# Processive ATP-driven Substrate Disassembly by the *N*-Ethylmaleimide-sensitive Factor (NSF) Molecular Machine

## 

**Files in this Data Supplement:**

- Supplemental (.pdf, 438 KB) - A single supplemental figure for publication online with the manuscript.
