# Supplementary material for: Processive ATP-driven Substrate Disassembly by the N-Ethylmaleimide-sensitive Factor (NSF) Molecular Machine
Source: J Biol Chem. 2013 Jun 17;288(32):23436–45. doi: 10.1074/jbc.M113.476705 (PMC4520572; doi:10.1074/jbc.M113.476705)
Supplement: Supplemental Data [file supp_M113.476705_jbc.M113.476705-1.pdf]

SUPPLEMENTAL MATERIAL

Figure S1. **Amino acid sequences of expressed protein constructs used in this study.** Underlined sequences show the TEV cleavage sites used to cleave of the N-terminal His-tags show in bold. In the case of the GCN4 tetramer, the N-terminal tag was maltose binding protein. Cysteine residues used to label proteins with Oregon Green 488 Maleimide are highlighted in green. SNARE core domains are colored in red. In the case of the ext-SNARE complex repeats of the SNARE core domain are colored in blue. The repeated sequence of the SNAP-25 linker is shown in italics.

Figure S1

Soluble SNARE complex:

Syntaxin$_{1-265}$ C145S S249C K253C:
1 MKDRTQELRT AKDSDDDDDV TVTVDRDRFM DEFFEQVEEI RGFIDKIAEN VEEVKRKHSA
61 ILASPNPDEK TKEELEELMS DIKKTANKVR SKLKSIEQSI EQEEGLNRSS ADLRIRKTQH
121 STLSRKFVEV MSEYNATQSD YRERSKGRIQ RQLEITGRTT TSEELEDMLE SGNPAIFASG
181 IIMDSSISKQ ALSEIETRHS EIIKLENSIR ELHDMFMDMA MLVESQGEMI DRIEYNVEHA
241 VDYVERAVCD TKCAVKYQSK ARRKK

Synaptobrevin$_{1-96}$:
1 **MASYYHHHHH HDYDIPTSEN LYFQ**GASHMS ATAATVPPAA PAGEGGPPAP PPNLTSNRRL
61 QQTQAQVDEV VDIMRVNVDK VLERDQKLSE LDDRADALQA GASQFETSAA KLKRKYWWKN
121 LKMM

SNAP-25:
1 MASMAEDADM RNELEEMQRR ADQLADESLE STRRMLQLVE ESKDAGIRTL VMLDEQGEQL
61 DRVEEGMNHI NQDMKEAEKN LKDLGKCCGL FICPCNKLKS SDAYKKAWGN NQDGVVASQP
121 ARVVDEREQM AISGGFIRRV TNDARENEMD ENLEQVSGII GNLRHMALDM GNEIDTQNRQ
181 IDRIMEKADS NKTRIDEANQ RATKMLGSG

Ext-SNARE complex:

Ext-Syntaxin:
1 MKDRTQELRT AKDSDDDDDV TVTVDRDRFM DEFFEQVEEI RGFIDKIAEN VEEVKRKHSA
61 ILASPNPDEK TKEELEELMS DIKKTANKVR SKLKSIEQSI EQEEGLNRSS ADLRIRKTQH
121 STLSRKFVEV MSEYNATQSD YRERSKGRIQ RQLEITGRTT TSEELEDMLE SGNPAIFASG
181 IIMDSSISKQ ALSEIETRHS EIIKLENSIR ELHDMFMDMA MLVESQGEMI DRIEYNVEHA
241 VDYVERAVSD TKKAVKEIIK LENSIRELHD MFMDMAMLVE SLGEMIDRIE YNVEHAVDYV
301 ERAVCDTKCA VKYQSKARRK K

Ext-Synaptobrevin:
1 **MASYYHHHHH HDYDIPTSEN LYFQ**GASHMS ATAATVPPAA PAGEGGPPAP PPNLTSNRRL
61 QQTQAQVDEV VDIMRVNVDK VLERDQKLSE LDDRADALQA GASQFETSAA KLKRKLQQTQ
121 AQVDEVVDIM RVNVDKVLEA DQKLSELDDR ADALQAGASQ FETSAAKLKR KYWWKNLKMM

Ext-SNAP-25:
1 MAEDADMRNE LEEMQRRADQ LADESLESTR RMLQLVEESK DAGIRTLVML DEQGEQLDRV
61 EEGMNHINQD MKEAEKNLKD LGKSTRRMLQ LVEESKDAGI RTLVMLDEAG EQLDRVEEGM
121 NHINQDMKEA EKNLKDLGKC CGLFICPCNK LKSSDAYKKA WGNNQDGVVA SQPARVVDER
181 EQMAISGGFI RRVTN*GLFIS PSNKLKSSDA YKKAWGNNQD GVVASQPARV VDEREQMAIS*
241 *GGFIRRVTND* ARENEMDENL EQVSGIIGNL RHMALDMGNE IDTQNRQIDR IMEKADSNKT
301 RIDEANQRAT KMDENLEQVS GIIGNLRHMA LDMGNEIDTL NRQIDRIMEK ADSNKTRIDE
361 ANQRATKMLG SG

Mini SNARE complex:

Syntaxin$_{180-262}$ S249C K253C:
1 MGIIMDSSIS KQALSEIETR HSEIIKLENS IRELHDMFMD MAMLVESQGE MIDRIEYNVE
61 HAVDYVERAV CDTKCAVKYQ SKAR

Synaptobrevin$_{2-96}$:
1 **MASYYHHHHH HDYDIPTSEN LYFQ**GASHMS ATAATVPPAA PAGEGGPPAP PPNLTSNRRL
61 QQTQAQVDEV VDIMRVNVDK VLERDQKLSE LDDRADALQA GASQFETSAA KLKRKYWWKN
121 LKMM

SNAP-25$_{1-85}$:
1 MAEDADMRNE LEEMQRRADQ LADESLESTR RMLQLVEESK DAGIRTLVML DEQGEQLDRV
61 EEGMNHINQD MKEAEKNLKD LGKCC

SNAP-25$_{120-206}$:
1 MVVDEREQMA ISGGFIRRVT NDARENEMDE NLEQVSGIIG NLRHMALDMG NEIDTQNRQI
61 DRIMEKADSN KTRIDEANQR ATKMLGSG

GCN4 tetramer:
1 **MGSHHHHHHS GKIEEGKLVI WINGDKGYNG LAEVGKKFEK DTGIKVTVEH PDKLEEKFPQ**
61 **VAATGDGPDI IFWAHDRFGG YAQSGLLAEI TPDKAFQDKL YPFTWDAVRY NGKLIAYPIA**
121 **VEALSLIYNK DLLPNPPKTW EEIPALDKEL KAKGKSALMF NLQEPYFTWP LIAADGGYAF**
181 **KYENGKYDIK DVGVDNAGAK AGLTFLVDLI KNKHMNADTD YSIAEAAFNK GETAMTINGP**
241 **WAWSNIDTSK VNYGVTVLPT FKGQPSKPFV GVLSAGINAA SPNKELAKEF LENYLLTDEG**
301 **LEAVNKDKPL GAVALKSYEE ELAKDPRIAA TMENAQKGEI MPNIPQMSAF WYAVRTAVIN**
361 **AASGRQTVDE ALKDAQTNSS SSGTASGGAT TSENLYFQ**GH MRLKQIEDKL EEILSKLYHI
421 ENELARIKKL LGEILDRLKQ IEDKLEEILS KLYHIENELA RIKCLLGEIL E

NSF:
1 MGMAGRSMQA ARCPTDELSL SNCAVVSEKD YQSGQHVIVR TSPNHKYIFT LRTHPSVVPG
61 SVAFSLPQRK WAGLSIGQEI EVALYSFDKA KQCIGTMTIE IDFLQKKNID SNPYDTDKMA
121 AEFIQQFNNQ AFSVGQQLVF SFNDKLFGLL VKDIEAMDPS ILKGEPASGK RQKIEVGLVV
181 GNSQVAFEKA ENSSLNLIGK AKTKENRQSI INPDWNFEKM GIGGLDKEFS DIFRRAFASR
241 VFPPEIVEQM GCKHVKGILL YGPPGCGKTL LARQIGKMLN AREPKVVNGP EILNKYVGES
301 EANIRKLFAD AEEEQRRLGA NSGLHIIIFD EIDAICKQRG SMAGSTGVHD TVVNQLLSKI
361 DGVEQLNNIL VIGMTNRPDL IDEALLRPGR LEVKMEIGLP DEKGRLQILH IHTARMRGHQ
421 LLSADVDIKE LAVETKNFSG AELEGLVRAA QSTAMNRHIK ASTKVEVDME KAESLQVTRG
481 DFLASLENDI KPAFGTNQED YASYIMNGII KWGDPVTRVL DDGELLVQQT KNSDRTPLVS
541 VLLEGPPHSG KTALAAKIAE ESNFPFIKIC SPDKMIGFSE TAKCQAMKKI FDDAYKSQLS
601 CVVVDDIERL LDYVPIGPRF SNLVLQALLV LLKKAPPQGR KLLIIGTTSR KDVLQEMEML
661 NAFSTTIHVP NIATGEQLLE ALELLGNFKD KERTTIAQQV KGKKVWIGIK KLLMLIEMSL
721 QMDPEYRVRK FLALLREEGA SPLDFDTAAL EHHHHHH

αSNAP:
1 **MHHHHHHHHH HENLYFQ**GMD TSGKQAEAMA LLAEAERKVK NSQSFFSGLF GGSSKIEEAC
61 EIYARAANMF KMAKNWSAAG NAFCQAAQLH LQLQSKHDAA TCFVDAGNAF KKADPQEAIN
121 CLMRAIEIYT DMGRFTIAAK HHISIAEIYE TELVDVEKAI AHYEQSADYY KGEESNSSAN
181 KCLLKVAGYA AQLEQYQKAI DIYEQVGTSA MDSPLLKYSA KDYFFKAALC HFCIDMLNAK
241 LAVQKYEELF PAFSDSRECK LMKKLLEAHE EQNVDSYTES VKEYDSISRL DQWLTTMLLR
301 IKKTIQGDEE DLR
